# Odor and VOC Emissions from Pan Frying of Mackerel at Three Stages: Raw, Well-Done, and Charred

**DOI:** 10.3390/ijerph111111753

**Published:** 2014-11-14

**Authors:** Jeong-Hyeon Ahn, Jan E. Szulejko, Ki-Hyun Kim, Yong-Hyun Kim, Bo-Won Kim

**Affiliations:** Department of Civil and Environmental Engineering, Hanyang University, Seoul 133-791, Korea; E-Mails: qq112311@naver.com (J.-H.A.); kkim61@hanyang.ac.kr (K.-H.K.); inocent01@nate.com (Y.-H.K.); samsuet@nate.com (B.-W.K.)

**Keywords:** mackerel, odorant, volatile organic compounds (VOC), sorbent tube, GC-MS

## Abstract

Many classes of odorants and volatile organic compounds that are deleterious to our wellbeing can be emitted from diverse cooking activities. Once emitted, they can persist in our living space for varying durations. In this study, various volatile organic compounds released prior to and during the pan frying of fish (mackerel) were analyzed at three different cooking stages (stage 1 = raw (R), stage 2 = well-done (W), and stage 3 = overcooked/charred (O)). Generally, most volatile organic compounds recorded their highest concentration levels at stage 3 (O), e.g., 465 (trimethylamine) and 106 ppb (acetic acid). In contrast, at stage 2 (W), the lowest volatile organic compounds emissions were observed. The overall results of this study confirm that trimethylamine is identified as the strongest odorous compound, especially prior to cooking (stage 1 (R)) and during overcooking leading to charring (stage 3 (O)). As there is a paucity of research effort to measure odor intensities from pan frying of mackerel, this study will provide valuable information regarding the management of indoor air quality.

## 1. Introduction

In recent years, most people spend an increasing amount of time indoors or in enclosed spaces, to an extent of >90% per day at home, work, or in vehicles (5.5%) [1]. With the increasing time spent indoors, there have been growing concerns about indoor air quality (IAQ). Numerous volatile organic compounds (VOCs) with various functional groups and odor strengths have been identified in indoor environments. Many countries worldwide have passed and enacted various IAQ regulatory laws on permissible VOC concentrations and/or requirements for the improved ventilation. The sources of odorant emissions in an enclosed space are very diverse to include cooking, garbage, smoking, toilet odor, interior materials, outgassing from furniture and construction materials, biogenic, *etc.* [2,3,4,5]. Cooking and food storage can release many odorous compounds such as sulfurous, nitrogeneous, volatile fatty acid, aldehyde, hydrocarbon, and alcohol compounds [6]. Chronic exposure to indoor air pollutants can induce various symptoms such as headache, fatigue, dermatitis, *etc.* These presenting symptoms have been commonly used to diagnose Sick Building Syndrome (SBS) which stimulated considerable public interest about the air quality of indoor environments [7].

It is reported that many compounds emitted during cooking in a house or enclosed space (especially with poor ventilation) can impact human health in various ways [8]. The options to maintain a pleasant IAQ include: masking with air deodorants, natural ventilation, or HVAC (heating, ventilating, and air conditioning) systems, their effectiveness or efficiencies are often not satisfactory [9]. Among the pollutants released during cooking, aldehydes are some of the most potent eye and skin irritants. Especially during food preparation, the level and type of VOCs emissions are strongly affected by a combination of variables such as: food ingredients, cooking oil, heating fuels, and cooking practices being employed. Apart from strong odorants, other hazardous pollutants (like carcinogens) are also emitted at different cooking stages [10]. These compounds can significantly impact human health, e.g., skin problems, headaches, respiratory diseases, *etc.* In case of lung cancer in women non-smokers, a number of factors (e.g., cooking practices and frequency) are one of the main causes in addition to other well-known factors (e.g., second-hand smoke) [11].

The aims of this research were to study the speciation of VOCs (and odorants) and to measure their concentrations before and during pan-frying of mackerel over a portable butane fuelled cooker. Based on this analysis, the emission characteristics of odors and VOCs were estimated. In our activities of daily living (ADL) depending on the cooking style, cooking can be the major source of pollutant emissions hazardous to human health [12,13]. In the preliminary work for this study, the reproducibility of IAQ parameters was investigated intensively in a series of replicate experiments on the pan-frying of mackerel [14]. Accordingly, the total VOC emissions (TVOC) can be measured at higher confidence, as reported previously in our replicate cooking experiments (*n* = 11). To learn more about the odor impact of pan-frying mackerel, a closed room indoor pollution study was done at three cooking stages (raw, well-cooked, and charred). The results of this fish frying study can offer valuable insights into odor and VOC emissions.

## 2. Experimental Section 

For the analysis of various VOC emitted at different cooking stages, samples were collected by frying mackerel on a pan over a butane fueled portable cooker simulating home-cooking. Air samples at each cooking stage were collected and analyzed. The collected samples were analyzed by GC-MS. For quantification, liquid/gaseous phase standards were prepared containing a total of 23 target compounds. These 23 target compounds were used to make quantitative predictions for compounds lacking authentic standards or surrogates (CLASS) using the carbon number concept and method [15,16].

### 2.1. Preparation of Working Standards (WS)

Information on calibration and basic QA/QC is essential to obtain reliable data in the analysis of airborne VOCs. For the quantitation of VOCs emitted from fish frying, liquid working standards were prepared containing a total of 22 VOCs, *i.e.*, aldehydes, ketones, aromatic hydrocarbons, volatile fatty acids, and an amine (Table 1). However, in the exceptional case of TMA, gaseous standards were used.

**Table 1:** Basic information on target (and reference) compounds detected at three different cooking stages of fish (mackerel) samples.

Order	Group	Compound	Short Name	MW (g/mol)	Formula	CAS Number
**A. Target Compounds**						
1	Aldehydes	Propionaldehyde	PA	58.1	C_3_H_6_O	123-38-6
2		Butyraldehyde	BA	72.1	C_4_H_8_O	123-72-8
3		Isovaleraldehyde	IA	86.1	C_5_H_10_O	590-86-3
4		Valeraldehyde	VA	86.1	C_5_H_10_O	110-62-3
5		Methyl ethyl ketone	MEK	72.1	C_4_H_8_O	78-93-3
6	Ketones	Methyl isobutyl ketone	MIBK	100	C_6_H_12_O	108-10-1
7		Butyl acetate	BuAc	116	C_6_H_12_O_2_	123-86-4
8		Isobutyl alcohol	*i*-BuAl	74.1	C_4_H_10_O	78-83-1
9	Aromatic	Benzene	B	78.1	C_6_H_6_	71-43-2
10	hydrocarbons	Toluene	T	92.1	C_7_H_8_	108-88-3
11		*p*-Xylene	*p*-X	106	C_8_H_10_	106-42-3
12		*m*-Xylene	*m*-X	106	C_8_H_10_	108-38-3
13		*o*-Xylene	*o*-X	106	C_8_H_10_	95-47-6
14		Styrene	S	104	C_8_H_8_	100-42-5
15	Volatile	Acetic acid	ACA	60.1	C_2_H_4_O_2_	64-19-7
16	fatty acids	Propionic acid	PPA	74.1	C_3_H_6_O_2_	79-09-4
17		*i*-Butyric acid	IBA	88.1	C_4_H_8_O_2_	79-31.2
18		*n*-Butyric acid	BTA	88.1	C_4_H_8_O_2_	107-92-6
19		*i*-Valeric acid	IVA	102	C_5_H_10_O_2_	503-74-2
20		*n*-Valeric acid	VLA	102	C_5_H_10_O_2_	109-52-4
21		Hexanoic acid	HXA	116	C_6_H_12_O_2_	142-62-1
22		Heptanoic acid	HPA	130	C_7_H_14_O_2_	111-14-8
23	Amine	Trimethylamine	TMA	59.1	C_3_H_9_N	75-50-3
**B. Reference Compounds**						
1	Aliphatic	*n*-Decane	--	142	C_10_H_22_	124-18-5
2	Hydrocarbons	*n*-Dodecane	--	170	C_12_H_26_	112-40-3
3	Aldehydes	Crotonaldehyde	--	70.1	C_4_H_6_O	4170-30-3
4		*n*-Hexanaldehyde	--	100	C_6_H_12_O	66-25-1
5		*n*-Heptanal	--	114	C_7_H_14_O	111-71-7
6		*n*-Octanaldehyde	--	128	C_8_H_16_O	124-13-0
7		(E,E)-2,4-Decadienal	--	152	C_10_H_16_O	25152-84-5
8		trans-2-Decenal	--	154	C_10_H_18_O	3913-71-1
9		2-Undecenal	--	168	C_11_H_20_O	2463-77-6
10	Alcohol	1-Pentanol	--	88.2	C_5_H_12_O	71-41-0
11	Haloalkane	Chloroform	--	119	CHCl_3_	67-66-3

Reagent grade chemicals (RGC) with purities >97% were purchased (Sigma-Aldrich, St Louis, MO, USA) to prepare liquid phase standards of 22 target compounds (except trimethylamine, Rigas, Daejeon, Korea). Liquid working standards (L-WS) were made by the diluting RGCs in a stepwise manner in methanol. Concentrations of the L-WSs for a four-point calibration were in the range 4.91–49.1 ng (in case of benzene, see Table 2). The calibration of these four-point L-WS was conducted at a fixed standard volume (FSV) method [17]. In case of TMA, 1002 ppb gaseous working standard (G-WS) was prepared by mixing the primary standard (5010 ppm) with nitrogen (99.999%). For a four-point calibration of the TMA G-WS, different volumes (24.2–242 ng of TMA) were analyzed using the fixed standard concentration (FSC) approach [17] (Table 2).

### 2.2. The Collection of Odorants from Fish Frying

**Figure 1:**
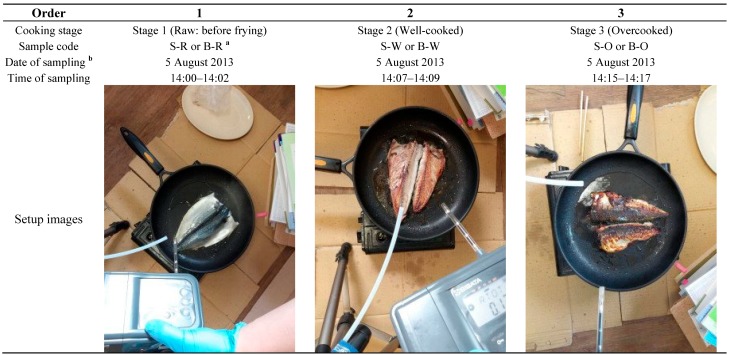
Basic information on mackerel frying conditions for the sample collection (310 g) in this study.

This research used an unfrozen filleted mackerel (see Figure 1) that was shipped from Jeju Island, South Korea the previous day and purchased from a local market. Mackerel is in high demand in South Korea and can be easily cooked. The initial weight of the mackerel (fillet) before frying was approximately 310 g. Experiments were started approximately 1 h after purchase of the mackerel fillet. Mackerel fillet was pan fried using a portable butane fuelled cooker without the addition of any cooking oil to eliminate its confounding emissions. Butane is the most commonly used fuel for portable cookers in South Korea. As an oily fish, mackerel releases its own oil during cooking.

In the preliminary study, the reproducibility of the IAQ metric was ascertained from replicate pan frying of eleven mackerel fillets in a room (94.5 m^3^ volume with dimensions 6.0 m × 6.3 m × 2.5 m (height)) [14]. However, in this study, the experiments were conducted in a smaller room (42.9 m^3^ volume with dimensions 4.4 m × 3.9 m × 2.5 m (height)). In both the present and preliminary experiments, the ventilation in room was maintained at minimal level by the HVAC system. More specifically, all windows and the door were kept closed throughout sampling except briefly soon after completion of sampling when the samples were transferred to the analytical laboratory for analysis. The room temperature was kept between 23 to 27 °C with a relative humidity (RH) of 75%.

**Table 2:** Preparation of standards for 23 target compounds (22 in liquid- and one (TMA) in gas-phase).

**A-1. Preparation of primary standard and working standard of 22 compounds**																								
	**Compound**	**PA**	**BA**	**IA**	**VA**	**MEK**	**MIBK**	**BuAc**	**i-BuAl**	**B**	**T**	**p-X**	**m-X**	**o-X**	**S**	**ACA**	**PPA**	**IBA**	**BTA**	**IVA**	**VLA**	**HXA**	**HPA**	**Methanol**
Primary Grade	Purity (%)	97.0	99.0	97.0	97.0	99.0	99.5	99.5	99.0	99.5	99.5	99.0	99.0	97.0	99.0	99.99	99.0	99.0	99.0	99.0	99.0	99.0	99.0	--
Chemical	Density (g/mL)	0.798	0.805	0.797	0.81	0.805	0.802	0.881	0.801	0.878	0.866	0.87	0.87	0.88	0.906	1.049	0.99	0.9697	0.96	0.93	0.94	0.927	0.9181	--
Primary	Volume (μL)	300	300	300	300	300	300	300	300	300	300	300	300	300	300	300	300	300	300	300	300	300	300	13,400
Standard	Concentration (ng/μL)	11,611	11,954	11,596	11,786	11,954	11,970	13,149	11,895	13,104	12,925	12,845	12,845	12,804	13,454	15,733	14,702	14,400	14,226	13,736	19,482	13,766	13,634	--
1st WS	Volume (μL)	--	--	--	--	--	--	--	--	--	--	300	--	--	--	--	--	--		--	--	--	--	19,700
--	Concentration (ng/μL)	174	179	174	177	179	180	197	178	197	194	193	193	192	202	236	221	216	213	206	292	206	205	--
**A-2. Preparation of (final) liquid working standard for four point calibration ^a^**																								
**Order**	**Mixing Volume (μL)**		**Mass (ng) in 1 µL**																					
	**1st WS**	**Methanol**	**PA**	**BA**	**IA**	**VA**	**MEK**	**MIBK**	**BuAc**	**i-BuAl**	**B**	**T**	**p-X**	**m-X**	**o-X**	**S**	**ACA**	**PPA**	**IBA**	**BTA**	**IVA**	**VLA**	**HXA**	**HPA**
1	40	1560	4.35	4.48	4.35	4.42	4.48	4.49	4.93	4.46	4.91	4.85	4.82	4.82	4.80	5.05	5.90	5.51	5.40	5.33	5.15	7.31	5.16	5.11
2	80	1520	8.71	8.97	8.70	8.84	8.97	8.98	9.86	8.92	9.83	9.69	9.63	9.63	9.60	10.1	11.8	11.0	10.8	10.7	10.3	14.6	10.3	10.2
3	160	1440	17.4	17.9	17.4	17.7	17.9	18.0	19.7	17.8	19.7	19.4	19.3	19.3	19.2	20.2	23.6	22.1	21.6	21.3	20.6	29.2	20.6	20.5
4	400	1200	43.5	44.8	43.5	44.2	44.8	44.9	49.3	44.6	49.1	48.5	48.2	48.2	48.0	50.5	59.0	55.1	54.0	53.3	51.5	73.1	51.6	51.1
**B-1. Preparation of the 1st working standard of TMA (gas phase)**																								
			**Compound**							**TMA**							**N_2_**							
Primary Standard			Concentration (ppm)							5010							99.999%							
1st WS			Volume (mL)							0.2							999.8							
			Concentration (ppb)							1002							--							
**B-2. Adsorbed mass (ng) of TMA for four point calibration**																								
**Order**			**Loading**							**Loading**							**TMA**							
			**time^a^ (min)**							**volume (mL)**							**mass (ng)**							
1			0.1							10							24.2							
2			0.2							20							48.4							
3			0.5							50							121							
4			1							100							242							

**^a^** Total volume of 1.6 mL for standard of each concentration level.

**^a^** Sample code: sampling method (sorbent tube or bag)—cooking status ^**b**^ Mackerel was purchased at 13:00 and kept for 1 h at 2 °C in fridge.

To collect VOCs released from either raw or frying mackerel, samples for VOC analysis were collected at varying frying states such as (1) stage 1 = raw (R): started immediately once placed on the frying pan, (2) stage 2 = well-done (W): what people usually eat, and (3) stage 3 = overcooked (O): with the mackerel surface completely charred, *i.e.*, unfit and unpleasant to eat (Figure 1). This one-time experiment was conducted to discern changes in VOCs emissions with cooking stages under the same room conditions.

Odorants released from all three different stages of fish samples were captured using both bag and sorbent tube (ST) samplers. Bag sampling was conducted with a lung sampler (ACEN Co. Ltd., Seoul, Korea) into a 10 L polyester aluminum (PEA) bag. Polluted air was sampled by bag sampling method (1 m above the frying pan) via at Teflon (PTFE) tubing (1/4” OD × 1/8” ID × 1.5 m) to simulate the approximate position of the cook’s nose. Paired samples were collected simultaneously by bag and sorbent tube for comparison purposes. For analysis, the collected air samples in the PEA bag were pulled through an ST tube at a flow rate of 100 mL·min^−1^ for 2 min. At the same sampling height, a 3-bed quartz ST packed with (1) Carbopack C (100 mg), (2) Carbopack B (70 mg), and (3) Carbopack × (70 mg) was also positioned for ST sampling [18]. At all three frying stage, the ST collection of airborne VOCs was made at a flow rate of 100 mL·min^−1^ for 2 min. The recovery factor of the sampling medium does not need to be explicitly known as it is folded into the response factor as discussed in references [16] and [19]; hence, accurate VOC quantitation is assured. The reliability of this 3-bed ST setup has been validated recently by us [16].

### 2.3. Instrumental Setup for Analysis

For calibration, the L-WS was loaded into one end of ST with a microsyringe (through a temporary injection site pierced into the PTFE tube) into a constant sweeping N_2_ flow at 100 mL·atm·min^−1^ for 2 min. The ST loaded with target VOCs was analyzed by thermal desorption (TD; Markes International, Ltd, Unity, Rhondda Cynon Taff, UK) and gas chromatography–mass spectrometry (GC-MS; Shimadzu, GCMS-QP2010, Kyoto, Japan).

After an ST was loaded in the TD unit, analytes were thermally desorbed at 300 °C for 10 min to transfer analytes to the cold trap (CT) maintained at −5 °C in the TD unit. The CT was packed with Carbopack C and Carbopack B in a 1:1 volume ratio. Afterwards, the analytes were desorbed off the CT at 320 °C for 10 minutes and parked on CP-Wax GC column (0.25 mm ID × 60 m, 0.25 µm film thickness), The GC oven program was initialized at 40 °C for 10 minutes and ramped to 220 °C at 5 °C·min^−1^ for a total 50 min analytical run time (Table 3).

## 3. Results and Discussion

### 3.1. Calibration Characteristics

In order to quantify the VOCs released from the frying of mackerel, the basic calibration and QA experiments were conducted on 23 target VOCs by ST/TD-GC-MS. Response factors (RF) of target VOCs ranged from 7828 (propionaldehyde) to 178,910 (*o*-xylene). The *R*^2^ values of calibration for all target compounds were satisfactory at >0.99. The results of relative standard error (RSE, %), if used as a measure of the reproducibility of analytic technique, were <5% (range from 0.07% (*p*-xylene) to 4.14% (acetic acid)). RSE was computed by triplicate measurements on the 3rd calibration point (Table 2). Method detection limits (MDL) were determined by heptaplicate analysis of the 0.08 ng·µL^−1^ L-WS. MDL ranged from 0.006 ng (butyraldehyde) to 0.108 ng (propionaldehyde). When applied to a 200 mL sample volume, the corresponding MDL in ppb was between 0.007 (*p*-xylene) and 0.228 (propionaldehyde).

**Table 3:** The TD-GC-MS instrumental settings employed for the analysis of VOCs and odorants from fish cooking in this work.

**a. Sampling conditions**			
Sampling flow rate:	100 mL·min^−1^	Sorbent tube sampling temperature:	~70 °C
Sampling volume:	200 mL	Bag sampling temperature:	25 °C
**b. Sorbent tube desorption settings**			
Sorbent material:	Carbopack C + Carbopack B + Carbopack X (mass = 100, 70, 70 mg)		
Desorption flow:	50 mL·min^−1^		
Desorption time:	10 min	Desorption temperature:	300 °C
**c. Thermal desorber (Unity, Markes International, Ltd.) settings**			
Cold trap sorbent:	Carbopack C + Carbopack B (volume ratio = 1:1)		
Split ratio:	1:5	Adsorption temperature:	−5 °C
Split flow:	5 mL	Desorption temperature:	320 °C
Trap hold time:	10 min	Flow path temperature:	180 °C
**d. GC (Shimadzu GC-2010) and Q MS (Shimadzu GCMS-QP2010) settings**			
Column: CP Wax (diameter: 0.25 mm, length: 60 m, and film thickness: 0.25 µm)			
Oven settings		MS detector settings	
Oven temperature:	40 °C (10 min)	Ionization mode:	EI (70 eV)
Oven ramping rate:	5 °C·min^−1^	Ion source temperature:	230 °C
Max oven temperature:	220 °C (4 min)	Interface temperature:	230 °C
Total run time:	50 min	TIC scan range:	35~600 m/z
Carrier gas:	He (99.999%)	Emission current:	150 μA
Carrier Pressure:	25.0 psi		

**Figure 2:**
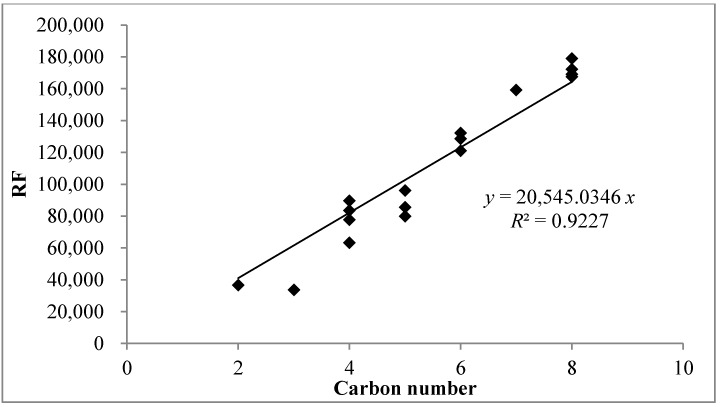
Predictive equation based on carbon number approach for quantification of non-target (reference) compounds.

Out of the 23 target VOCs, only 11 VOCs were frequently detected in the fish frying air samples analyzed by GC-MS. For the non-target and reference VOCs, their RF values were estimated using predictive equations (Figure 2) based on the carbon number (CN) concept using the RF values of a training set comprising of 23 target compounds [16]. The *R*^2^ value of the predictive equation showed satisfactory linearity (0.9227). As a result, the RF of chloroform with the lowest carbon number (CN = 1) was predicted to be 20,545, whereas the RF of n-dodecane with the largest carbon number (CN = 12) was predicted to be 246,540. It should be noted that the RF of chloroform is probably ~60,000 which is about 1/3 for that of xylene (~180,000) based on the work of Szulejko and Kim [20]. The predicted *RF* values > 180,000 are unrealistic, as those values are far above the maximum instrument intrinsic *RF* of ~180,000 (*i.e.*, bias due to sorbent tube breakthrough is minimal) in this experiment [19]. However, in this study, it needs reasonably good estimates for RF for both target compounds and reference compounds. Hence, the estimated RF values above 180,000 were used as is through the approximation based on the carbon number concept. The calculated MDLs of the non-target compounds (11 VOCs) based on predictive RF *vs.* CN correlation ranged from 0.026 ng (*n*-dodecane) to 0.318 ng (chloroform). For a 200 mL sample volume, the predicted MDLs corresponded to 0.004 to 0.065 ppb, respectively (Table 4).

### 3.2. Comparison of Concentrations between Bag Sampling and Sorbent Tube Sampling Method

In this research, two different sampling methods (bag *vs.* sorbent tube sampling method) were compared. One of the sampling comparative factors to consider is the possible bias due to the high air temperature (in the vicinity of the sampling point). In case of bag sampling method, the end of sniffer line is heated by the convection plume from the BBQ. However, the bag (B) being remote from the sampling point is near room temperature (25 °C) and sample losses in the PTFE sniffer line is expected to be minimal [21]. In contrast, the temperature of the sorbent tube (S) was as high as (~70 ± 5 °C) and variable; this may lead to sorbent bed breakthrough for the lighter VOCs (e.g., acetaldehyde and propionaldehyde (not detected)).

**Figure 3:**
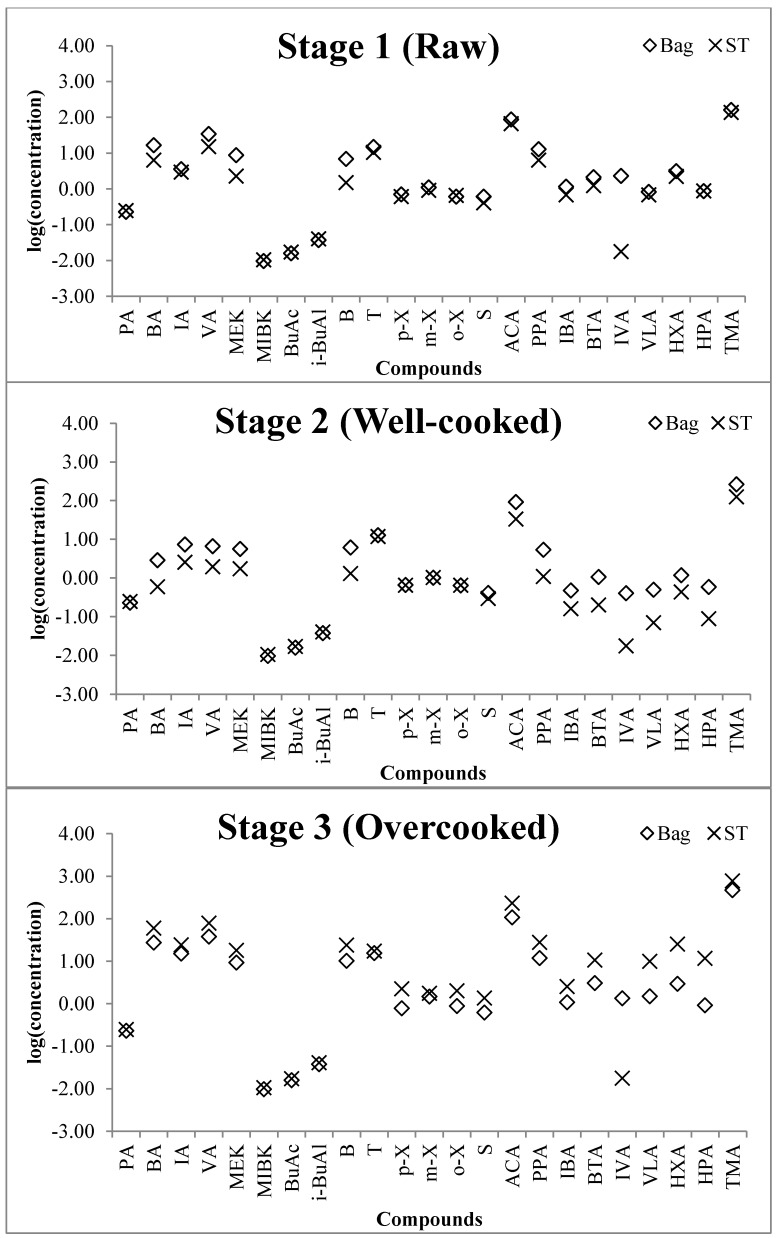
Comparison of concentration (ppb) of all compounds between three different cooking stages.

Inspection of the (S/B) ratios of VOCs in Table 5 showed fair to good agreement (>0.67 and ≤1) at stage 1 (R) when the ST temperature was near room temperature and hence reduced breakthrough. Both sampling methods gave similar VOC concentration results. However, at stage 2 (W), most of the compounds (S/B) ratios were low showing negative bias except aromatic compounds (toluene, *p-*, *m*-, *o*-xylene, and styrene, *i.e.*, not expected to exhibit breakthrough). The lowest ratio was *n*-valeric acid (0.14) in stage 2 (W) and the highest ratio was heptanoic acid (12.5) in stage 3 (O). The S/B ratio of toluene, one of the aromatic compounds (having large breakthrough volume at 70 °C), was 0.70 (stage 1 (R)), 0.91 (stage 2 (W)), and 1.11 (stage 3 (O)). This implies that VOC concentrations determined by the bag sampling approach were higher than sorbent tube sampling approach, although the aromatic compounds showed the least relative bias due to the minimal breakthrough. On the other hand, most of the compounds measured in stage 3 (O) showed positive bias (sorbent tube *vs.* bag sampling) (Figure 3). Consequently, the temperature in the vicinity of the sorbent tube during frying is suspected to be the main cause of biases when sampling VOCs. Because of noticeable ST biases under this study conditions, the bag sampling data are discussed in greater detail than the ST data for in-depth analysis of VOCs released from fish frying at different stages unless specified otherwise.

**Table 4:** Results of ST-TD-GC-MS based-calibration of VOC and the basic QA parameters determined in this study: comparison of response factor (RF), *R*^2^, and relative standard error (RSE, %) and method detection limit (MDL, ng and ppb).

Order	Group	Compound	RF	*R*^2^	RSE ^a^ (%)	MDL	
						(ng)	(ppb) ^b^
**A. Target compounds**							
1	Aldehydes	Propionaldehyde	7828	0.9923	3.01	0.108	0.228
2		Butyraldehyde	63,131	0.9927	0.63	0.006	0.011
3		Isovaleraldehyde	95,981	0.9960	0.89	0.010	0.015
4		Valeraldehyde	79,889	0.9957	3.18	0.026	0.037
5		Methyl ethyl ketone	83,474	0.9938	0.87	0.037	0.063
6	Ketones	Methyl isobutyl ketone	132,143	0.9974	0.91	0.008	0.010
7		Butyl acetate	128,506	0.9924	1.27	0.015	0.016
8		Isobutyl alcohol	89,544	0.9962	0.83	0.023	0.037
9		Benzene	120,914	0.9925	1.23	0.011	0.017
10		Toluene	159,105	0.9932	1.05	0.034	0.045
11	Aromatic	*p*-Xylene	172,208	0.9905	0.07	0.006	0.007
12	hydrocarbons	*m*-Xylene	167,521	0.9925	1.56	0.015	0.017
13		*o*-Xylene	178,910	0.9911	1.13	0.019	0.022
14		Styrene	169,088	0.9921	1.82	0.017	0.020
15		Acetic acid	36,696	0.9919	4.14	0.036	0.072
16		Propionic acid	33,591	0.9954	2.47	0.039	0.064
17		*i*-Butyric acid	75,064	0.9910	1.26	0.017	0.023
18	Volatile	*n*-Butyric acid	77,611	0.9944	1.86	0.017	0.023
19	fatty acids	*i*-Valeric acid	96,075	0.9958	0.72	0.014	0.016
20		*n*-Valeric acid	85,543	0.9952	0.55	0.015	0.018
21		Hexanoic acid	85,823	0.9971	0.13	0.015	0.016
22		Heptanoic acid	87,487	0.9968	1.09	0.015	0.014
23	Amine ^c^	Trimethylamine	27,952	0.9988	--	0.047	0.097
**B. Reference compounds ^c^**							
1	Aliphatic	*n*-Decane	205,450	--	--	0.032	0.005
2	hydrocarbons	*n*-Dodecane	246,540	--	--	0.026	0.004
3		Crotonaldehyde	82,180	--	--	0.079	0.028
4		*n*-Hexanaldehyde	123,270	--	--	0.053	0.013
5	Aldehydes	*n*-Heptanal	143,815	--	--	0.045	0.010
6		*n*-Octanaldehyde	164,360	--	--	0.040	0.008
7		(*E,E*)-2,4-Decadienal	205,450	--	--	0.032	0.005
8		*trans*-2-Decenal	205,450	--	--	0.032	0.005
9		2-Undecenal	225,995	--	--	0.029	0.004
10	Alcohol	1-Pentanol	102,725	--	--	0.064	0.018
11	Haloalkane	Chloroform	20,545	--	--	0.318	0.065

^a^ Triplicate analyses of the final WS (corresponding to the 3rd calibration point); ^b^ For the calculation of MDL (in ppb), sample volume of 200 mL was assumed; ^c^ Calibration was made by gaseous standard.

### 3.3. The Evaluation of Odorants Emitted from Mackerel at Different Frying Stages

In this research, the mackerel cooking states were classified according to the extent of frying. A total of 19 VOCs out of all 23 target VOCs (except four VOCs: PA, MIBK, BuAc, and *i*-BuAl) were detected in all environmental samples (Table 5A). In case of TMA, the well-known cause of fishy smell [22,23] was observed at 160 and 265 ppb at stage 1 (R) and stage 2 (W), respectively. In the present work, the highest concentration was observed at stage 3 (O) at 465 ppb. ACA was 86.7, 92.5, and 106 ppb and VA was also 34.4, 6.61, and 37.7 ppb (bag sampling) at each stage.

By applying the RFs values estimated from the predictive equation based on carbon number [16,24], the 11 reference VOCs were quantified as explained above. The 11 reference VOCs consisted of aliphatic hydrocarbons (*n* = 2), aldehydes (*n* = 7), an alcohol (*n* = 1) and one other (*n* = 1). It is noteworthy that many of the reference compounds in the aldehyde group were detected at relatively high concentrations. In stage 1 (R), crotonaldehyde and *n*-hexanaldehyde recorded approximately 51.7 and 46.3 ppb, respectively.

Inspection of the overall patterns of emitted compounds showed that their values at stage 1 (R) and 2 (W) had almost similar profiles. However, the concentrations at stage 3 (O) tended to peak sharply. Consequently, the trend of pollutant emissions was seen on the order of stage 3 (O) > 1 (R) ≥ 2 (W) in that order. Additionally, in our recent study on the reproducibility of pollutants released from cooking [14], aldehydes, acetaldehyde, isovaleraldehyde, and valeraldehyde averaged 99.3, 3.96 and 12.2 ppb, respectively. Compared with this experiment, concentration especially in stage 3 (O) of isovaleraldehyde and valeraldehyde concentraions were higher than previous research.

### 3.4. The Evaluation of Odor Intensity at Each Frying Stage

**Figure 4:**
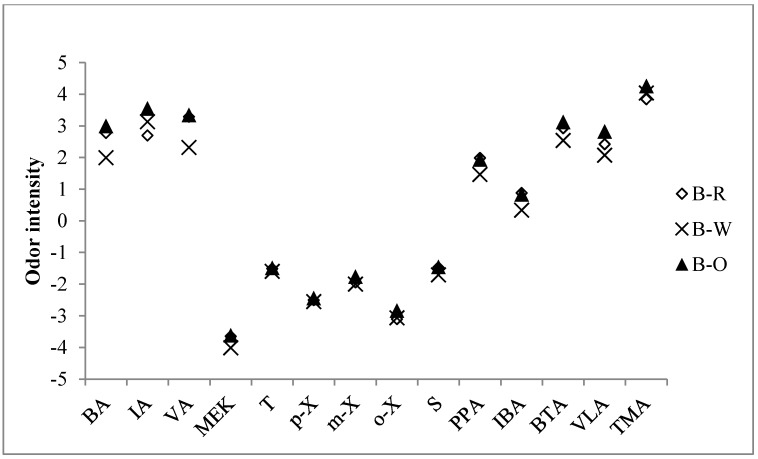
Comparison of odor intensity (OI) values of target compounds measured at different cooking stages (bag sampling).

Of the available approaches for assessing and interpreting odor strength of VOCs and odorants, we selected the odor intensity (OI) concept. For this assessment, concentration of 16 odorants measured in this work was converted into OI based on the related formula proposed by [25] (Figure 4). The sum of odor intensity (SOI) for comprehensive comparisons (with the literature) was also calculated from OI of each compound as follows (Equation (1)):

SOI = log (10OI(1) + 10OI(2) + … + 10OI(n))
(1)

Using this formula, SOI values calculated at each cooking stage were 4.05, 4.12, and 4.43. In line with the general expectation, the highest SOI was observed at stage 3 (O) overcooked. Inspection of the OI values for each compound showed that TMA was consistently larger at all stages 3.84 (stage 1 (R)), 4.04 (stage 2 (W)), and 4.26 (stage 3 (O)). From this result, TMA was by far the dominant compound contributing to the SOI. On the other hand, PA and MIBK being below the MDL made no contribution to SOI (Table 6).

Prior work on TMA emissions from mackerel gave an OI value of ~5.25 [26]. The OI value of TMA in this work averaged 4.00 (stage 1 (R) = 3.84, stage 2 (W) = 4.04, stage 3 (O) = 4.26) and was relatively lower than previously reported. TMA is the most potent odorant of all analyzed target compounds in this work. TMA is also a significant source of malodor in both not only in fresh fish but also in overcooked fish. Analysis of various environmental samples reported in the literature revealed that there are many other compounds in food stuffs contributing to the SOI. For example, sulfur compounds (H_2_S, CH_3_SH, DMS, DMDS) are major malodorants emitted when roasting coffee (SOI = 6.42) and from boiled egg (SOI = 4.09) [27,28]. In addition, SOI of roasting coffee beans and frying cabbage recorded 6.50 and 4.52, respectively [29].

**Table 5:** Concentration (ppb) and ratio between the two sampling methods (sorbent tube/bag (S/B)) of VOCs measured from gases samples collected at each frying stage.

Order	Group	Compound	Concentration (ppb) by Bag Method at Each Stage			Concentration (ppb) by Sorbent Tube Method at Each Stage			Ratio (S/B) of VOCs		
			B-R ^a^	B-W	B-O	S-R	S-W	S-O	R	W	O
**A. Target compounds**											
1	Aldehydes	Propionaldehyde	0.228	0.228	0.228	0.247	0.247	0.247	--	--	--
2		Butyraldehyde	16.6	2.88	27.1	6.36	0.60	59.6	0.38	0.21	2.20
3		Isovaleraldehyde	3.53	7.34	15.0	2.92	2.56	24.1	0.83	0.35	1.61
4		Valeraldehyde	34.4	6.61	37.7	15.1	1.97	78.2	0.44	0.30	2.07
5		Methyl ethyl ketone	8.81	5.61	9.16	2.28	1.75	17.8	0.26	0.31	1.94
6	Ketones	Methyl isobutyl ketone	0.010	0.010	0.010	0.011	0.011	0.011	--	--	--
7		Butyl acetate	0.016	0.016	0.016	0.017	0.017	0.017	--	--	--
8		Isobutyl alcohol	0.037	0.037	0.037	0.041	0.041	0.041	--	--	--
9		Benzene	6.83	6.14	10.1	1.49	1.31	23.9	0.22	0.21	2.37
10		Toluene	14.9	12.8	15.5	10.5	11.7	17.2	0.71	0.92	1.11
11	Aromatic	*p*-Xylene	0.71	0.66	0.78	0.61	0.65	2.24	0.86	0.98	2.89
12	hydrocarbons	*m*-Xylene	1.10	1.02	1.47	0.90	1.04	1.76	0.82	1.02	1.20
13		*o*-Xylene	0.60	0.64	0.88	0.66	0.66	1.99	1.09	1.03	2.27
14		Styrene	0.61	0.41	0.62	0.41	0.30	1.36	0.67	0.72	2.21
15		Acetic acid	86.7	92.5	106	66.7	33.3	232	0.77	0.36	2.18
16		Propionic acid	12.8	5.37	11.7	6.39	1.10	27.5	0.50	0.20	2.34
17		*i*-Butyric acid	1.15	0.48	1.07	0.68	0.16	2.51	0.59	0.33	2.35
18	Volatile	*n*-Butyric acid	2.15	1.06	3.04	1.24	0.20	10.5	0.58	0.19	3.46
19	fatty acids	*i*-Valeric acid	2.30	0.40	1.34	0.018	0.018	0.018	-	-	-
20		*n*-Valeric acid	0.83	0.50	1.48	0.68	0.07	9.9	0.82	0.14	6.69
21		Hexanoic acid	3.15	1.18	2.93	2.22	0.44	24.9	0.71	0.37	8.51
22		Heptanoic acid	0.87	0.59	0.92	0.88	0.09	11.5	1.01	0.15	12.56
23	Amine	Trimethylamine	160	265	465	137	126	772	0.86	0.47	1.66
**B. Reference compounds**											
1	Aliphatic	*n*-Decane	1.46	0.97	2.33	1.00	0.93	4.29	0.69	0.96	1.84
2	hydrocarbons	*n*-Dodecane	0.88	0.42	1.13	0.19	0.09	1.94	0.21	0.20	1.72
3		Crotonaldehyde	28.0	4.96	51.7	12.3	0.90	111	0.44	0.18	2.14
4		*n*-Hexanaldehyde	46.3	10.3	42.7	21.5	2.95	90.0	0.46	0.29	2.11
5	Aldehydes	*n*-Heptanal	13.4	2.28	18.3	6.76	0.79	45.4	0.50	0.35	2.48
6		*n*-Octanaldehyde	7.46	2.44	10.5	3.94	0.51	32.0	0.53	0.21	3.06
7		(*E,E*)-2,4-Decadienal	5.54	1.01	3.96	9.38	0.45	31.3	1.69	0.45	7.92
8		*trans*-2-Decenal	14.7	2.14	13.5	11.2	0.84	62.9	0.76	0.39	4.65
9		2-Undecenal	5.85	1.10	5.07	9.29	0.56	50.7	1.59	0.51	10.00
10	Alcohol	1-Pentanol	24.7	4.50	19.3	12.8	1.65	68.0	0.52	0.37	3.52
11	Haloalkane	Chloroform	8.74	49.9	55.1	18.0	63.4	53.2	2.06	1.27	0.96

**^a^** Bag sampling (B), Sorbent tube sampling (S)—Stage 1 = raw (R), 2 = well-cooked (W), 3 = overcooked (O).

**Table 6:** Odor intensity (OI) formula of each compound and sum of odor intensity (SOI).

Order	Group	Compound	OI formula^a^	OI		
				B-R	B-W	B-O
1	Aldehydes	Propionaldehyde	Y = 1.010logX + 3.86	ND	ND	ND
2		Butyraldehyde	Y = 1.030logX + 4.61	2.78	1.99	3.00
3		Isovaleraldehyde	Y = 1.350logX + 6.01	2.70	3.13	3.55
4		Valeraldehyde	Y = 1.360logX + 5.28	3.29	2.32	3.34
5	Ketones	Methyl ethyl ketone	Y = 1.850logX + 0.149	−3.65	−4.01	−3.62
6		Methyl isobutyl ketone	Y = 1.650logX + 2.27	ND	ND	ND
7		Toluene	Y = 1.400logX + 1.05	−1.51	−1.60	−1.49
8	Aromatic	*p*-Xylene	Y = 1.570logX + 2.44	−2.51	−2.56	−2.44
9	hydrocarbons	*m*-Xylene	Y = 1.460logX + 2.37	−1.95	−2.00	−1.77
10		*o*-Xylene	Y = 1.660logX + 2.24	−3.10	−3.07	−2.84
11		Styrene	Y = 1.420logX + 3.10	−1.47	−1.71	−1.46
12		Propionic acid	Y = 1.380logX + 4.60	1.99	1.47	1.94
13	Volatile	*i*-Butyric acid	Y = 1.430logX + 5.08	0.88	0.33	0.83
14	fatty acids	*n*-Butyric acid	Y = 1.290logX + 6.37	2.93	2.53	3.12
15		*n*-Valeric acid	Y = 1.580logX + 7.29	2.42	2.07	2.82
16	Amine	Trimethylamine	Y = 0.901logX + 4.56	3.84	4.04	4.26
**SOI^c^**			--	4.05	4.12	4.43

^a^ Refer to Nagata [25] X: concentration (ppm), and Y: odor intensity; ^b^ Not calculated; ^c^ SOI = log(10^OI_(1) _+ 10^OI_(2)_ + … + 10^OI_(n)_).

## 4. Conclusions

In this work, various VOCs released during the pan frying of fish (mackerel) were analyzed at three different levels of cooking steps. We collected environmental air samples into sorbent tube and bag samplers for VOC quantification. The samples collected by the sorbent tube method were subject to large bias (both positive and negative) relative to the bag samples due to the sorbent been exposed to high unregulated air temperatures. The principal odorant emissions were generally in this order: stage 3 (O) > stage 1 (R) ≥ stage 2 (W). Of the emitted VOCs from fish frying, TMA is especially a potent source of odor and recorded its highest concentration of 465 ppb at stage 3 (O). Similarly, ACA was also recorded its highest concentration (106 ppb) at stage 3 (O). If the present work’s OI data are compared. TMA appears to be by far the largest contributor to the SOI at all stages. Therefore, TMA is an important odorant released from both fresh and overcooked fish. The concentrations of the reference 11 VOCs along with the 23 target compounds were calculated using the carbon number concept. A number of aldehyde compounds were detected exceeded the maximum regulatory IAQ limits in these experiments. The highest VOCs concentrations were found at stage 3 (O). The degree of fish frying simultaneously increased both the emission of odorants and VOCs.

The results of this study clearly suggest that various volatiles released from cooking activities should have a very major impact on human malodor perception, so adequate ventilation is an important factor for indoor air quality control when cooking inside [30]. Many types of hazardous compounds against to our wellbeing are emitted in massive quantities during cooking activities and can persist to cause the potential chronic diseases. Thus, we hope that this research outputs can contribute to a better understanding on the serious one of such issues and provide some basic tactics to control indoor air quality.

## Abbreviations

ACA: acetic acid
ADL: activities of daily living
B: benzene
BA: butyraldehyde
BTA: *n*-butyric acid
BuAc: butyl acetate
CLASS: compounds lacking authentic standards or surrogates
CN: carbon number
CT: cold trap
FSC: fixed standard concentration
FSV: fixed standard volume
GC: gas chromatography
HPA: heptanoic acid
HVAC: heating, ventilating, and air conditioning
HXA: hexanoic acid
IA: *i*-valeraldehyde
IAQ: indoor air quality
IBA: *i*-butyric acid; *i*-BuAl; *i*-butyl alcohol
IVA: *i*-valeric acid
MDL: method detection limits
MEK: methyl ethyl ketone
MIBK: methyl *i*-butyl ketone
MS: mass spectrometry
PA: propionaldehyde
PEA: polyester aluminum
PM: particulate matter
PPA: propionic acid
ppb: parts per billion
QA: quality assurance
QC: quality control
RF: response factor
RGC: reagent grade chemical
RH: relative humidity
RSE: relative standard error
S: styrene
SBS: sick building syndrome
ST: sorbent tube
T: toluene
TD: thermal desorption
TMA: trimethylamine
TVOC: total VOC emissions
VA: valeraldehyde
VLA: *n*-valeric acid
VOC: volatile organic compound
WS: working standard
X: xylene
